# Template-assisted nanostructure fabrication by glancing angle deposition: a molecular dynamics study

**DOI:** 10.1186/1556-276X-8-312

**Published:** 2013-07-05

**Authors:** Junjie Zhang, Yongzhi Cao, Qiang Gao, Chao Wu, Fuli Yu, Yingchun Liang

**Affiliations:** 1 Center for Precision Engineering, Harbin Institute of Technology, Harbin 150001, People's Republic of China; 2 Nuclear and Radiation Safety Center, Ministry of Environmental Protection, Beijing 100082, People's Republic of China

**Keywords:** Glancing angle deposition, Template, Deformation mechanism, Molecular dynamics

## Abstract

In the present work, we investigate the pre-existing template-assisted glancing angle deposition of Al columnar structures on Cu substrate by means of molecular dynamics simulations, with a focus on examining the effect of deposition-induced template deformation on the morphologies of the fabricated structures. Our simulations demonstrate that the pre-existing templates significantly intensify the shadowing effect, which thus facilitates the formation of columnar structures under small deposition flux. The underlying deformation modes of the templates under different deposition configurations are analyzed and are correlated to the geometrical characteristics of the columnar structures. It is found that the template height-dependent deformation behavior of the templates strongly influences the morphologies of the fabricated columnar structures. Our findings provide design and fabrication guidelines for the fabrication of one-dimensional nanostructures by the template-assisted deposition technique.

## Background

One-dimensional (1D) nanostructures, including nanopillars, nanorods, nanotubes, and nanowires, are promising building blocks for constructing nanoscale electronical and optoelectronical elements and interconnects because of their unique physical properties [1]. In addition to the characterization, the fabrication of ordered arrays of 1D nanostructures has been one of the current research focuses of nanostructures engineering. In particular, the rotational glancing angle deposition (GLAD) has been demonstrated to be one powerful nanostructuring technique for the fabrication of columnar nanostructures in an orientation- and structure-controllable, material-independent fashion [2-6]. The rotational GLAD as a physical vapor deposition is extended from the static GLAD (oblique angle deposition) by adding azimuthal and/or polar rotations of the substrate. During the rotational GLAD process, the lateral component of deposition flux with respect to the surface normal of the substrate contributes to the formation of columnar structures due to the shadowing effect, while the rotation of the substrate eliminates the preferred orientation growth, thus controls the shape of the structures. In the past few decades, there is considerable effort of both experimental investigation and atomistic simulations taken to investigate the fundamental mechanisms of the rotational GLAD [7-11].

Since nucleated islands acting as shadowing centers are essentially required for the formation of columnar structures in the initial period of the rotational GLAD, recently placing nano-sized templates on the bare substrate is proposed to replace the nucleated islands, in such a way both deposition period and deposition flux can be reduced significantly. Most importantly, by designing the geometry and the alignment of the templates, ordered arrays of columnar structures with pre-designed shapes can be fabricated under the intensified shadowing effect [12,13]. Although the template-assisted rotational GLAD has been demonstrated to be one promising nanostructuring technique for the fabrication of 1D nanostructures, our fundamental understanding of the deposition process, particularly the deposition-induced deformation of the templates, is still limited: will the templates deform during the deposition? If yes, what are the underlying deformation mechanisms of the templates? And how does the deformation behavior of the templates influence the geometry of the fabricated columnar structures?

In this letter, we address the above questions by performing three-dimensional molecular dynamics (MD) simulations of the template-assisted rotational GLAD of 1D Al columnar structures on Cu substrate. Our simulations demonstrate that the presence of templates significantly intensifies the shadowing effect to form 1D columnar structures when deposition flux is small, as compared to the template-free rotational GLAD. Furthermore, the morphology of the fabricated columnar structures by the template-assisted rotational GLAD strongly depends on the deformation behaviors of the templates.

## Methods

**Figure 1:**
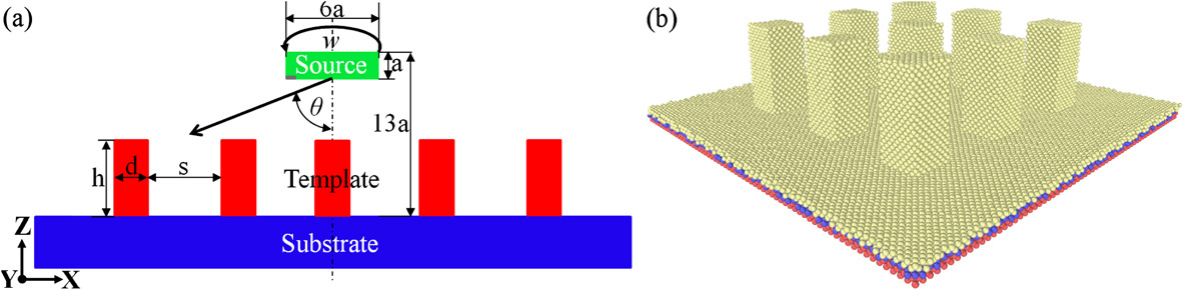
**MD model of the template-assisted rotational GLAD. (a)** Illustration of the deposition procedure; **(b)** atomic configuration of the substrate with pre-existing high templates. Atoms are colored according to their virtual types: red, blue, and yellow stand for boundary, thermostat, and mobile atoms, respectively.

Figure 1a illustrates the MD model of the template-assisted rotational GLAD utilized in the present work. The Cu substrate has a dimension of 11.6, 11.6, and 0.7 nm in *X*, *Y*, and *Z* directions, respectively. Periodic boundary condition (PBC) is imposed in the transverse *X* and *Y* directions of the substrate to simulate an infinitely wide thin film. There are nine equally spaced Cu templates of square cylinder placed on the substrate. The lattice constant *a* for Cu is 0.3615 nm. The width *d* for each template is 6*a*, and the distance *s* between each template is 10*a*. To investigate the influence of the template height *h* on the deposition process, two height values of 8*a* and 14*a* are considered. In addition, two other deposition configurations of template-free rotational GLAD and template-assisted static GLAD are also presented for comparison purpose. For simplicity, the four deposition configurations of template-free rotational GLAD, high template-assisted rotational GLAD, high template-assisted static GLAD, and low template-assisted rotational GLAD are referred to as NT-RGLAD, HT-RGLAD, HT-SGLAD, and LT-RGLAD, respectively. Figure 1b presents the atomic configuration of the Cu substrate with high templates, which contains three types of atoms: red stands for the boundary atoms fixed in space, blue indicates the thermostat atoms used for maintaining the temperature of the system to be constant value of 300 K, and yellow represents the mobile atoms which motion follows the Newton's second law of motion.

Prior to the deposition, the as-created substrates are first relaxed to their equilibrium configurations at 300 K by rescaling the velocities of the thermostat atoms. Then, the deposition is conducted by inserting single Al atom from the deposition source toward the Cu substrate surface along specific direction until 20,000 Al atoms are deposited. As shown in Figure 1a, the deposition source of cuboid shape has a dimension of 6*a*, 6*a*, and 1*a* in the *X*, *Y*, and *Z* directions, respectively. The coordinates of the Al atoms are randomly generated within the deposition source. For each case, the deposition rate, the incident energy, and the incident angle *θ* are the same as 5 atoms per picosecond, 0.1 eV, and 83°, respectively. To mimic the azimuthal rotation of the substrate during the rotational GLAD experiments, in current simulations the deposition source is rotated with a rotational velocity *w* of 100 ps^−1^. After the completion of the deposition processes, the Cu-Al systems are allowed to relax for 100 ps to reach their equilibrium configurations. More detailed description about the MD model can also be found elsewhere [14,15]. Table 1 lists the parameters employed in the four deposition configurations. The atomic interactions in the Cu-Al system are modeled by an embedded-atom method [16]. All the MD simulations are performed using the LAMMPS code with an integration time step of 1 fs [17]. To identify the deformation mechanisms of the substrate material, the technique of common neighbor analysis (CNA) is adopted, and the difference between twin boundary (TB) and intrinsic stacking fault (ISF) is further distinguished [18,19]. A single hexagonal close-packed (HCP) coordinated layer identifies a coherent TB, two adjacent HCP coordinated layers indicate an ISF, and two HCP coordinated layers with a FCC coordinated layer between them represent an extrinsic stacking fault (ESF). The Ovito and Atomeye are utilized together to analyze MD data and generate MD snapshots [20,21].

**Table 1:** Parameters for the four deposition configurations

**Configuration**	**Rotational velocity (ps**^**−1**^**)**	**Template geometry (*d*, *s*, *h*)**
NT-RGLAD	100	0, 0, 0
HT-RGLAD	100	6*a*, 10*a*, 14*a*
HT-SGLAD	0	6*a*, 10*a*, 14*a*
LT-RGLAD	100	6*a*, 10*a*, 8*a*

The *a* (0.3615 nm) is the lattice constant for Cu.

## Results and discussion

**Figure 2:**
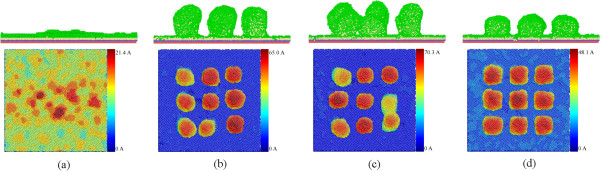
**Morphologies of the as-deposited nanostructures. (a)** Template-free rotational GLAD; **(b)** high template-assisted rotational GLAD; **(c)** high template-assisted static GLAD; **(d)** low template-assisted rotational GLAD. The upper row shows the front views, in which atoms are colored according to their virtual types: red, blue, and yellow stand for boundary, thermostat, and mobile atoms, respectively; the bottom row shows the top views, in which atoms are colored according to their heights.

Figure 2a presents the front and top views of the morphology of the Cu-Al system obtained after the template-free rotational GLAD, indicating that there is no columnar structure formed. The upper row of Figure 2a shows that the Al thin film grows in a layer-by-layer fashion on the Cu substrate, which is inconsistent with previous work [14,15]. However, there are islands formed on the surface of the formed Al thin film when the deposition flux is small. The islands resulting from the shadowing effect serves as shadowing centers to facilitate the formation of columnar structures during further GLAD deposition. Recent work suggests that low incident energy may significantly enhance the possibility of columnar structure formation during the template-free rotational GLAD [10]. In contrast, there are patterns of columnar structures formed during the template-assisted rotational GLAD or the static GLAD when templates are placed on the Cu substrate, as shown in Figure 2b,c,d. Furthermore, most of the impinging Al atoms are received by the templates. Therefore, it clearly indicates that the presence of the templates can significantly facilitate the formation of columnar structures because of the intensified shadowing effect, given the limited deposition flux. It should be noted that because of the presence of PBC in the transverse directions of the substrate, the distance between the edge templates is larger than that between the templates within the simulation box, which may lower the possibility of columnar structure formation.

Figure 2 also shows that the morphology of the columnar structures strongly depends on the parameters of the deposition configurations. Figure 2b shows that the height distribution of the columnar structures obtained through the high template-assisted rotational GLAD is not uniform, although the heights of the templates are the same. Furthermore, slight inclination of the axial of the columnar structures is observed. For the template-assisted static GLAD, the inclination is more pronounced than the template-assisted rotational GLAD, as shown in Figure 2b. In addition, the discrepancy between the heights of the columnar structures is pronounced, and the coalescence of columnar structures occurs. A comparison between Figure 2b,c shows that the template-assisted rotational GLAD leads to a lower but more uniform columnar structures than the template-assisted static GLAD, given the same height of the templates. As compared to the high template-assisted rotational GLAD, Figure 2d shows that the morphologies of the columnar structures obtained through the low template-assisted rotational GLAD are more uniform, as the structures are mainly straight and the heights are almost the same. We note that the morphology of the columnar structures may strongly depend on the rotational velocity, which determines the coverage of deposited Al atoms in conjunction with the deposition rate. It suggests that the height of the templates has strong influence on the morphology of the columnar structures obtained through the template-assisted rotational GLAD.

**Figure 3:**
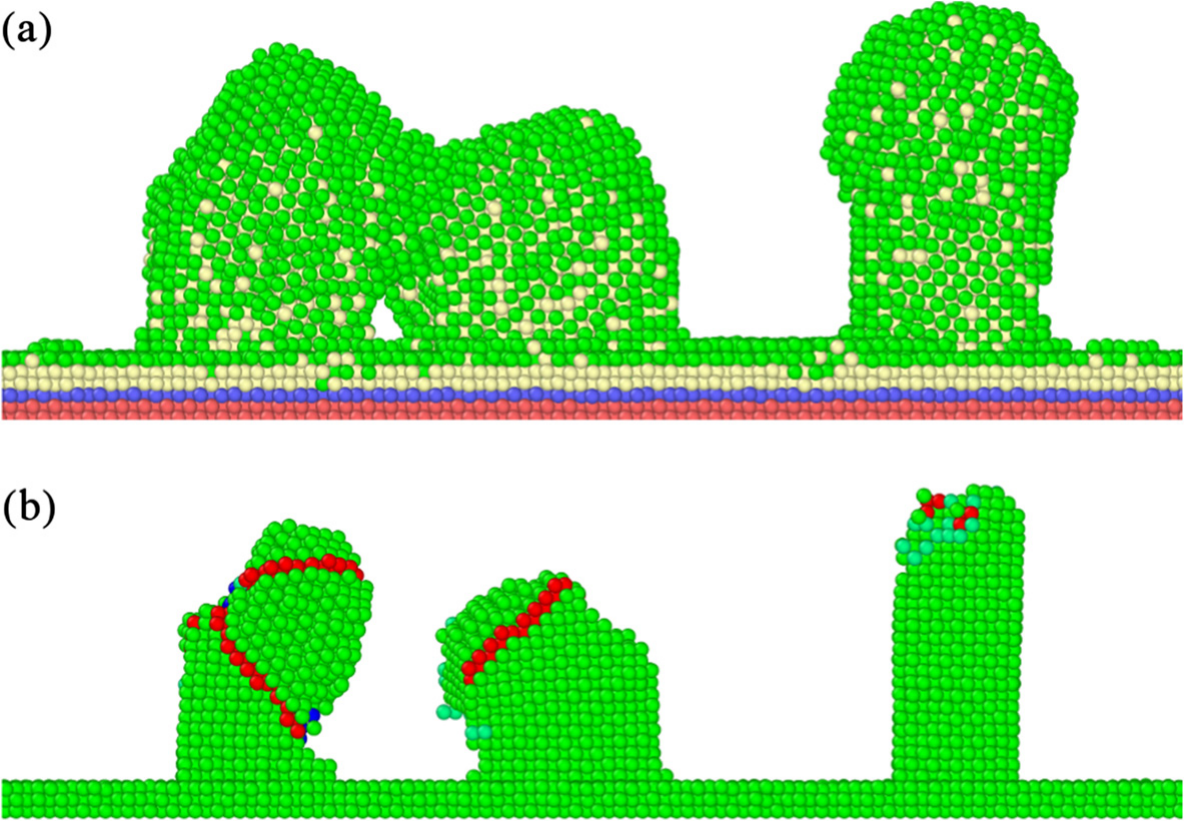
**Coalescence of columnar structures in template-assisted static GLAD. (a)** Enlarged view of the coalescence. Atoms are colored according to their types: red, blue, and yellow stand for boundary, thermostat, and mobile atoms, respectively; **(b)** defect configuration of the substrate shown in **(a)**. Atoms are colored according to their CNA values.

Figure 3a shows the enlarged view of the coalescence of the two columnar structures on the left side and in the middle obtained by the template-assisted static GLAD, which results from their inclination toward each other. The coalescence of columnar structures has also been reported by previous atomistic simulations [9,10]. In contrast, the columnar structure on the right side remains straight. To reveal the discrepancy between the morphologies of the columnar structures, defect analysis of the substrate including the templates is conducted. Figure 3b presents the defect configuration of the substrate shown in Figure 3a. The other atoms are eliminated to show defects clearly. In addition to the impact load applied by the impinging Al atoms, the local high temperature accompanied with the energy dissipation may also contribute to the formation of defects in the templates [22]. It is clearly seen from Figure 3b that there are two mechanical TBs inclining to each other formed in the template on the left side. The formation of mechanical TBs, i.e., deformation twinning, is an important deformation mode of 1D nanostructures with large surface-to-volume ratio under external load [23-25]. TB is a special kind of planar defects whose lattice structures exhibit mirror symmetries across the boundary. Therefore, the formation of TBs is accompanied with the change of the crystallographic orientation of the twin matrix. Consequently, the twinned part changes its shape with respect to the initial un-twinned one. The two inclined TBs in the template on the left side leads to more pronounced shape change than the template in the middle, in which there is only one TB formed. However, there is rather limited defect formed in the template on the right side.

**Figure 4:**
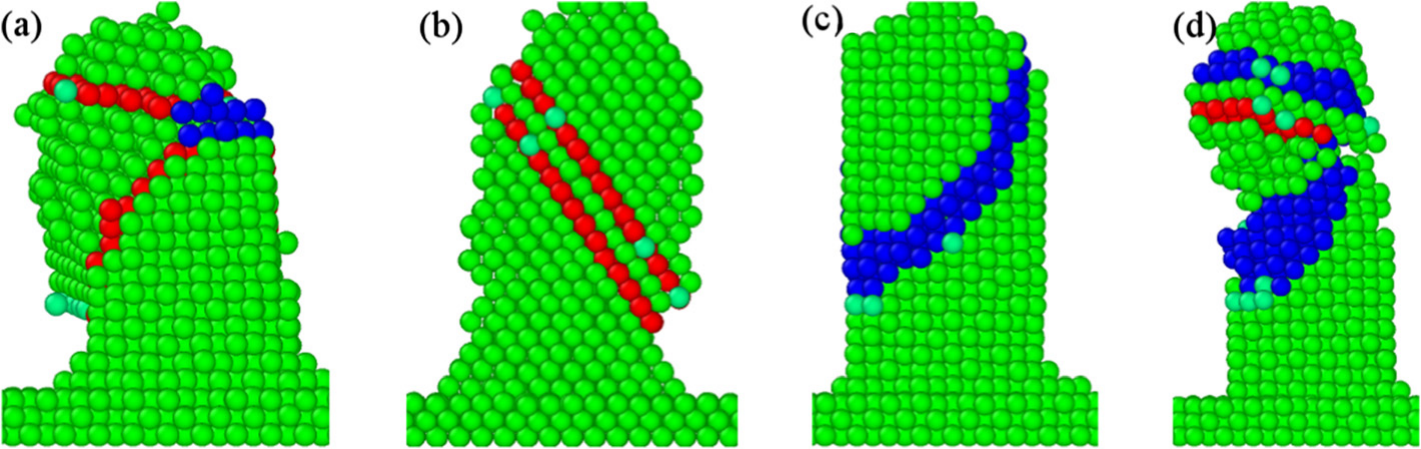
**Deformation mechanisms of the templates. (a)** Inclined TBs; **(b)** parallel TBs (ESF); **(c)** ISF; **(d)** mixing modes. Atoms are colored according to their CNA values.

In addition to deformation twinning, other deformation modes of the templates during deposition process are also investigated. Figure 4 presents representative deformation modes of the templates after the template-assisted rotational GLAD and static GLAD. Figure 4a shows that the deformation of the template is dominated by the formation of mechanical twins. The inclination of the two TBs leads to significant shape change of the template. Furthermore, Figure 4b demonstrates that when TBs are parallel to each other the shape change is less pronounced than that when TBs are inclined. In contrast to TBs that cause shape change of the templates, the formation of ISF only leads to shear of the upper part of the template by an atomic step, as demonstrated by Figure 4c. The defect structure presented in Figure 4b is an ESF, which originates from the dissociation of ISF [26]. Figure 4d presents the severe plastic deformation of the template, in which the dislocation mechanism and deformation twinning works in parallel. Furthermore, there is a neck region formed in the middle part of the template.

**Figure 5:**
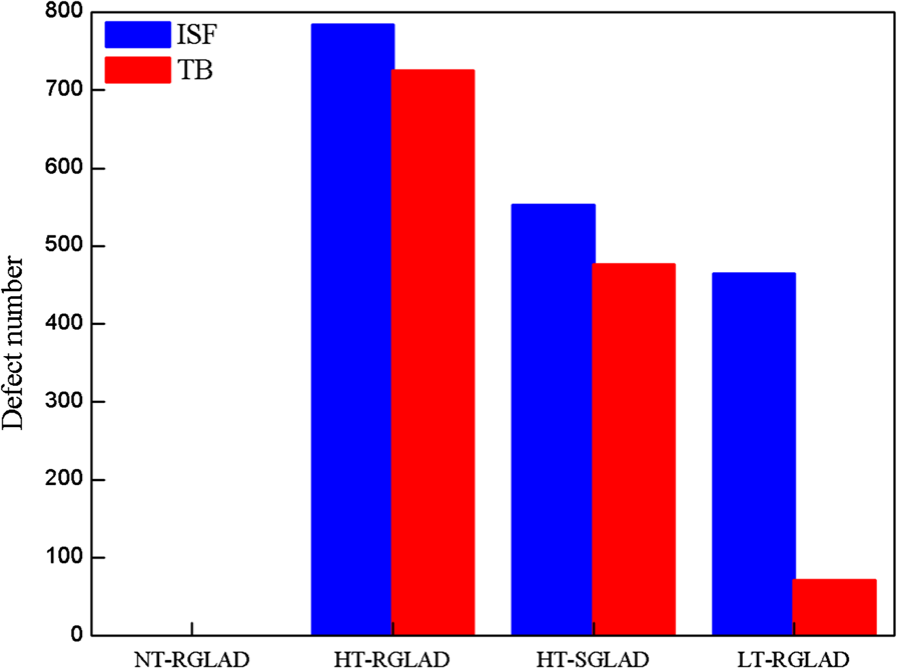
Number of defect atoms formed in the template after the deposition of four deposition configurations.

To quantitatively characterize the deformation mechanisms operating in the deformation of the templates, Figure 5 plots the number of ISF and TB atoms formed in the substrate after the depositions. It should be noted that the defects are analyzed based on the equilibrium configurations of the Cu-Al systems after the second relaxation. For the template-free substrate, the formed film is mainly in an amorphous state due to the small deposition flux, and there is neither ISF atom nor TB atom formed. In contrast, for the three template-assisted deposition processes, there are both ISF and TB atoms formed in the templates. Under the same height of the templates, both the number of ISF and TB atoms is larger for the rotational GLAD than that for the static GLAD. This may be attributed to the azimuthal rotation of the substrate during the rotational GLAD, which increases the contact area of the templates with impinging Al atoms. Figure 5 shows that both the number of ISF and TB atoms formed in the low template-assisted rotational GLAD is lower than that in the high template-assisted rotational GLAD. Furthermore, the reduction in the number of TB atoms is more pronounced than the ISF atoms, which implies that dislocation mechanisms is the main deformation mode of the low templates. The above results indicate that the deformation behavior of the templates dominates the morphology of the templates, which in turn influences the morphology of the columnar structures obtained through the template-assisted rotational GLAD or static GLAD.

## Conclusions

In summary, we perform MD simulations of the pre-existing template-assisted rotational GLAD to investigate the influence of templates on the formation of Al columnar nanostructures on Cu substrate. Our simulation results show that under small deposition flux, the presence of the templates significantly contributes to the formation of columnar structures due to the intensified shadowing effect, while there are only islands formed during template-free rotational GLAD. As compared to the template-assisted static GLAD, the azimuthal rotation of the substrate during the template-assisted rotational GLAD leads to uniform morphologies of the formed columnar structures. Our simulations reveal the two deformation modes of dislocation mechanisms and deformation twinning that operating in the plastic deformation of the templates, which strongly influence both the morphologies of the templates and the formed columnar structures. While the formation of TBs mainly causes the shape change of the templates, the presence of ISF leads to the shear of the template by an atomic step. Furthermore, the deformation modes dominating the plastic deformation of the templates change significantly with the height of the templates.

## Competing interests

The authors declare that they have no competing interests.

## Authors' contributions

JZ, YC, QG, and YL conceived the project. JZ, CW, and FY performed molecular dynamics simulations and analyzed data. JZ and YC wrote the paper. All authors read and approved the final manuscript.
